# Functional interplay between the Hippo pathway and heavy metals

**DOI:** 10.1080/23723556.2022.2061297

**Published:** 2022-04-09

**Authors:** Han Han, Alisa Mahieu, Lucas Dantas de Paula, Wenqi Wang

**Affiliations:** Department of Developmental and Cell Biology, University of California, Irvine, Irvine, CA, USA

**Keywords:** Hippo, MTF1, LATS, heavy metals

## Abstract

Emerging studies highlight the Hippo pathway as an important player in organ size control, tissue homeostasis, regeneration, development, and diseases, but our understanding of its roles and regulations remains incomplete. Our recent work reported a functional interplay between the Hippo pathway and heavy metals, providing new insights into this key signaling pathway.

The Hippo pathway has been established as a key regulator of organ size and tissue homeostasis.^1^ In mammals, the Hippo pathway components include Serine/Threonine kinases mammalian STE20-like protein kinase 1/2 (MST1/2) and large tumor suppressor kinase 1/2 (LATS1/2) as well as their adaptors protein Salvador homolog 1 (SAV1) and mob kinase activator 1 (MOB1), respectively. MST1/2 and their redundant kinases mitogen-activated protein kinase kinase kinase kinases (MAP4Ks) phosphorylate and activate LATS1/2, which in turn phosphorylate transcriptional co-activators yes-associated protein 1 (YAP) and transcriptional coactivator with PDZ-binding motif (TAZ), resulting in their cytoplasmic retention. When the Hippo pathway is inactivated, unphosphorylated YAP/TAZ are translocated into the nucleus to bind transcription factors TEA domain family members (TEADs), driving the expression of genes involved in proliferation and survival. Therefore, restricting YAP/TAZ has been considered the major functional output of the Hippo pathway.

Interestingly, our recent work revealed a YAP/TAZ-independent role of the Hippo pathway in heavy metal response. Heavy metals refer to metals or metalloids with relatively high density. Overloading of either essential heavy metals (e.g., zinc, copper, iron) or non-essential heavy metals (e.g., cadmium, lead) can cause severe cellular damage and even death.^2^ To counteract heavy metal toxicity, cells have developed a mechanism of producing Cysteine-rich peptides (e.g., metallothioneins) and heavy metal transporters to neutralize and export heavy metals, respectively.^3^ Such heavy metal response is regulated by metal regulatory transcription factor 1 (MTF1), the master regulator of heavy metal detoxification and homeostasis.^3^ Upon heavy metal load, zinc (Zn) directly binds MTF1 to induce its nuclear translocation; while, as for other heavy metals (e.g., cadmium, copper), they displace Zn from its-associated protein complexes to indirectly activate MTF1. However, detailed regulation of MTF1-dependent heavy metal response remains to be elucidated.

Our recent study connected the Hippo pathway with heavy metal homeostasis.^4^ Inhibition of the Hippo pathway protected cells from heavy metal toxicity both in cultured cells and in mice, which is independent of YAP and TAZ, but requires MTF1. Mechanistically, LATS1 interacted with and phosphorylated MTF1, resulting in the loss of its association with heavy metal response genes’ promoters and inhibition of heavy metal response genes’ transcription. Moreover, the Hippo pathway was inhibited in the heavy metal-treated cells and mouse tissues, a process mediated by the interaction between Zn and LATS1/2. Specifically, Zn directly bound LATS1/2 kinase domains and induced their conformational change, resulting in the loss of both LATS1/2 intrinsic kinase activities and their ability to be phosphorylated by MST1/2 and MAP4Ks. Thus, our work shed light on the molecular mechanism underlying heavy metal response, in which heavy metal load increases cellular Zn to bind both MTF1 and LATS1/2, so that MTF1 is able to enter the nucleus and bind the promoters of heavy metal response genes to promote their expression for heavy metal detoxification (Figure 1a).

**Figure 1.**
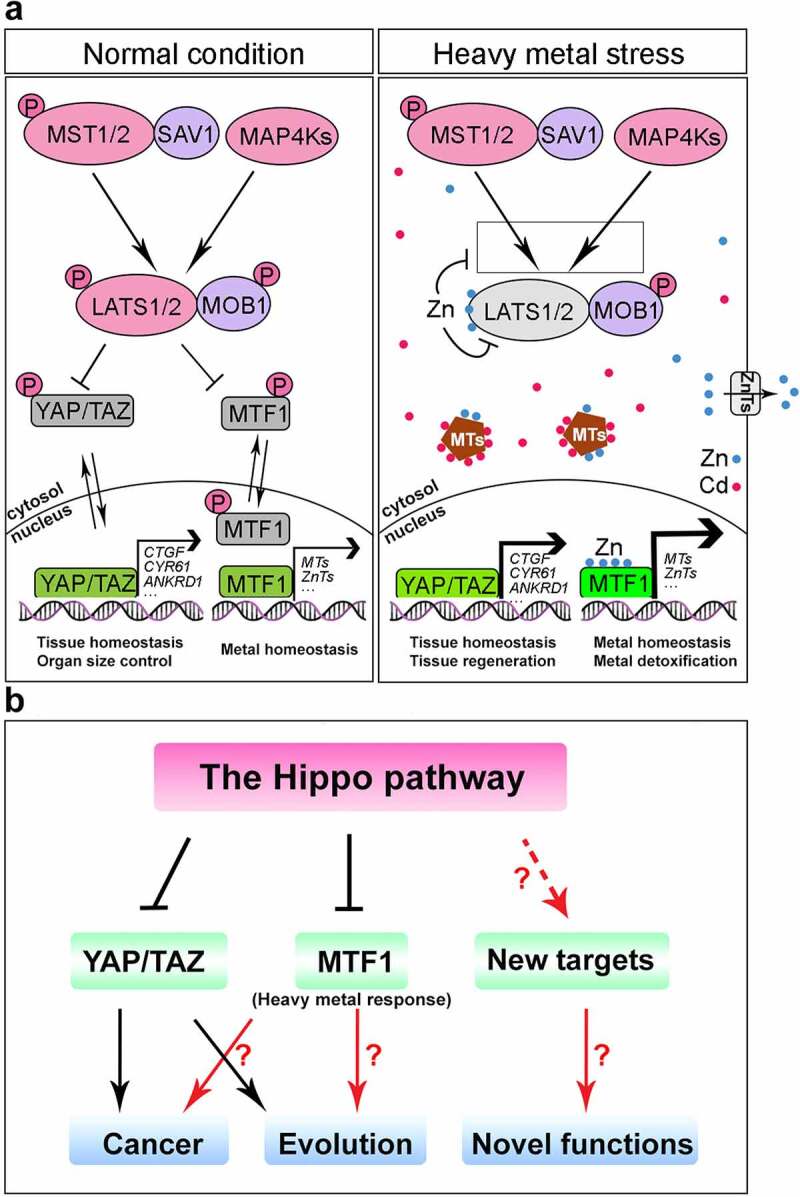
Regulation of the cellular response to heavy metals by the Hippo pathway.

(a) Under normal condition, the Hippo pathway kinases large tumor suppressor kinase 1/2 (LATS1/2) phosphorylate metal regulatory transcription factor 1 (MTF1) to inhibit its association with heavy metal response genes’ promoters, resulting in the loss of the expression of heavy metal response genes. Upon heavy metal load, elevated zinc (Zn) directly binds MTF1 to induce its nuclear localization; in the meantime, Zn physically interacts with and inhibits LATS1/2, allowing MTF1 to bind the promoters of heavy metal response genes and drive their transcription. Heavy metal response genes like metallothioneins (MTs) and heavy metal transporters like zinc transporters (ZnTs) can respectively neutralize and export overdosed heavy metals for detoxification and heavy metal homeostasis. MST1/2, STE20-like protein kinase 1/2; SAV1, protein Salvador homolog 1; MAP4Ks, mitogen-activated protein kinase kinase kinase kinases; MOB1, mob kinase activator 1; Cd, cadmium.

(b) It will be interesting to characterize the Hippo-mediated heavy metal response in the contexts of cancer development and evolution in future. Additional yes-associated protein 1 (YAP) and transcriptional coactivator with PDZ-binding motif (TAZ)-independent roles of the Hippo pathway also deserve further investigation.

However, key questions regarding this newly discovered function of the Hippo pathway in heavy metal response remain unanswered (Figure 1b).

First, what is the role of the Hippo-mediated heavy metal response in cancer development? Dysregulations of heavy metal homeostasis have been associated with multiple human diseases including cancers,^2^ while elevated MTF1 has also been observed in various types of cancer.^3^ These observations suggest that MTF1-dependent heavy metal response may be involved in Hippo pathway deficiency-induced tumorigenesis. We are now focusing on the Hippo-MTF1 axis in platinum (a type of heavy metals)-based chemotherapy. Platinum compounds such as cisplatin are effective chemotherapy drugs widely used for cancer treatment;^5^ however, cancer cells are prone to developing resistance to this treatment. Besides altered DNA repair and anti-apoptotic pathways, reasons accounting for cisplatin resistance also include the increased efflux of platinum compounds by heavy metal transporters and detoxification of bioactive platinum by metallothioneins,^5,6^ both of which are regulated by MTF1. Therefore, it will be interesting to determine whether the Hippo pathway can be employed as a chemo-sensitizer for platinum-related chemotherapy by controlling MTF1-dependent heavy metal response.

Second, how has evolution shaped the Hippo-mediated heavy metal response? We are interested in the evolutionary role of the Hippo pathway in heavy metal response, because **1**) the LATS1/2 phosphorylation site on MTF1 is evolutionarily conserved;^4^ **2**) functional Hippo pathway components can be traced to unicellular species;^1^ and **3**) MTF1 is evolutionarily conserved from insects to mammals^3^ and similar metal-responsive transcription factors have been identified in yeast.^3^ Although we have experimentally confirmed similar regulation of heavy metal response by the Hippo pathway in *Drosophila*,^4^ it is highly possible that such regulatory mechanisms exist in earlier species. What organisms first developed response mechanisms to heavy metals? When did MTF1 (or similar transcription factors) first function in this process? How did the Hippo pathway evolve to regulate MTF1? These are all key open questions regarding evolution that deserve further investigation.

Third, are there any other YAP/TAZ-independent functions of the Hippo pathway? Current Hippo functional studies are almost exclusively focused on YAP/TAZ-mediated cell proliferation and survival, while YAP/TAZ-independent roles have not been fully characterized at the same level. Interestingly, LATS1 phosphorylates angiomotoin (AMOT) family proteins to regulate cell migration and angiogenesis;^7,8^ MST1 phosphorylates interferon regulatory factor 3 (IRF3) to attenuate interferon production and antiviral defense;^9^ the Hippo upstream component neurofibromin-2 (NF2) binds DDB1 and CUL4 associated factor 1 (DCAF1) to suppress CRL4 E3 ubiquitin ligase activity.^10^ Although limited, these examples not only demonstrate that the Hippo pathway can modulate other cellular components in different signaling contexts, but also suggest that the Hippo pathway has a broader functional impact beyond growth control. Further characterizing the substrates of the Hippo kinases and the interactomes of the Hippo pathway components will provide novel insights into the YAP/TAZ-independent roles of this key signaling pathway.

## Data Availability

No experimental data were generated. Further information and requests for resources should be directed to corresponding author Dr. Wenqi Wang.
